# Promoting Rural-Residing Parents’ Receptivity to HPV Vaccination: Targeting Messages and Mobile Clinic Implementation

**DOI:** 10.3390/vaccines12070712

**Published:** 2024-06-26

**Authors:** Carla L. Fisher, M. Devyn Mullis, Antionette McFarlane, Marta D. Hansen, Melissa J. Vilaro, Carma L. Bylund, Lori Wiggins, Halie Corbitt, Stephanie A. S. Staras

**Affiliations:** 1 Department of Health Outcomes & Biomedical Informatics, College of Medicine, University of Florida, Gainesville, FL 32611, USA; mullis.md@ufl.edu (M.D.M.); m.hansen1@ufl.edu (M.D.H.); carma.bylund@ufl.edu (C.L.B.); sstaras@ufl.edu (S.A.S.S.); 2 Department of Emergency Medicine, College of Medicine, University of Florida, Gainesville, FL 32611, USA; amcfarlane@ufl.edu; 3 Department of Family, Youth and Community Sciences, Institute of Food and Agricultural Sciences, College of Agricultural and Life Sciences, University of Florida, Gainesville, FL 32611, USA; mgraveley@ufl.edu; 4 Institute of Food and Agricultural Sciences, College of Agricultural and Life Sciences, University of Florida, Gainesville, FL 32611, USA; lwiggins@ufl.edu (L.W.); hcorbitt@ufl.edu (H.C.)

**Keywords:** HPV vaccination, reminder messages, parents, mobile clinic, implementation

## Abstract

Interventions are needed to increase low HPV vaccination rates within rural areas in the United States, particularly in the state of Florida, which has the seventh highest number of HPV-related cancers. Florida also ranks low compared to other states in terms of HPV vaccination. Rural-residing parents may benefit from two evidence-based strategies to increase vaccination rates: reminder messages informing and prompting vaccination appointments and mobile clinics to reduce transportation barriers. We sought to identify parental attitudes towards (1) message features that promote rural-residing parents’ receptivity to HPV vaccination; (2) parents’ acceptability of three reminder message modalities (text, postcard, phone); and (3) implementation factors that promote parents’ acceptability of using a mobile clinic for vaccination. We recruited 28 rural-residing parents of 9- to 12-year-old children (unvaccinated for HPV) for focus group and individual interviews and thematically analyzed transcripts. Three features promoted parents’ receptivity to HPV vaccination messages: *source credibility*, *specific information coverage*, and *personalization (name and birthday wishes)*. Parents most preferred text messages and identified three factors promoting parents’ mobile clinic use: *convenience and feasibility, trustworthiness,* and *detailed information*. The findings indicate rural-residing parents’ acceptability of reminder messages and mobile clinics as well as the importance of trust and feasibility when implementing these evidence-based strategies for rural-residing parents.

## 1. Introduction

Human papillomavirus (HPV) vaccination has the potential to prevent 73% of HPV-related cancers including cervical, oropharyngeal, and vaginal cancers [1]. In the United States, HPV vaccination rates are well below recommended guidelines, both in terms of initiation rates (i.e., an individual receiving one or more dose) and up to date (UTD) rates (i.e., an individual completing the two doses when starting the series before age 15 or three doses when starting at 15 years of age or older) [2,3]. Given this, interventions are desperately needed to increase the unacceptably low HPV vaccination rates within the United States [2].

This need for HPV vaccination interventions is particularly true in the state of Florida, which currently has the seventh highest number of HPV-related cancers [4]. Florida ranks 35th of the 50 states in HPV vaccine initiation rate and 39th for overall UTD rate [2]. Similar to rural areas in the United States [2], the disparity is even greater within rural regions of Florida, including one subpopulation of rural Florida: the 11 rural North Central Florida counties (Rural-Urban Continuum Codes ≥ 4: Bradford, Columbia, Dixie, Hamilton, Lafayette, Levy, Madison, Putnam, Suwannee, Taylor, and Union), where the population is 8% Hispanic, 71% White, and 16% Black [5]. For instance, HPV-related cancer incidence is above the state of Florida’s average within 10 of these counties [6,7,8]. Furthermore, HPV vaccine initiation is below the state average for eight of these counties among 9- to 12-year-olds, which is the recommended target age for HPV vaccination by the American Cancer Society and American Academy of Pediatrics [6,7,8].

Interventions for increasing HPV vaccination rates with parents of unvaccinated children can be more impactful when culturally targeted using strategies that promote cultural appropriateness [9]. When the target culture or subpopulation (i.e., a defined group within a population with shared characteristics) informs the development of intervention materials and strategies, these resources can be responsive to that population’s distinct preferences, which reflect their priorities and values, thereby promoting their acceptability of the health information [10]. Thus, interventions that culturally target North Central Florida parents of unvaccinated 9- to 12-year-olds in these 11 rural counties could be more effective in promoting HPV vaccination initiation and UTD rates in this community. Parents whose adolescent has not received the HPV vaccine are primarily divided into two groups: intending and undecided/hesitant. Approximately 40% of parents *intend* to get the HPV vaccine for their adolescent in the next year but may not due to “not getting around to it” or “not seeing the doctor” [11,12]. On the other hand, 60% of parents who have not gotten the vaccine for their child are undecided or hesitant, and their hesitancy may be overcome with good communication [13,14].

In North Central Florida, rural-residing parents whose children have not received HPV vaccination may benefit from two evidence-based strategies for increasing vaccination rates: (1) reminder messages educating and prompting parents to make an appointment and (2) mobile clinics reducing transportation barriers [15,16]. Overwhelming evidence supports reminder messages as an evidence-based and recommended strategy for increasing HPV vaccine initiation [17,18,19,20,21,22,23]. While the findings are less consistent, reminder/recall increases the percentage of UTD adolescents and reduces the percentage of adolescents who are behind schedule on follow-up doses [22,24]. Additionally, mobile vaccination clinics are an acceptable and innovative way to bring preventive care to vulnerable communities, like rural-residing parents [25,26,27]. Mobile clinic vaccination options reduce transportation- and time-related challenges, which are known barriers to receiving the HPV vaccine for women and parents living in rural areas [28,29,30,31,32]. While these may be two optimal strategies for promoting parents’ initiation of HPV vaccination with North Central Florida rural parents of 9- to 12-year-olds, parents’ perspectives are needed prior to implementation to ensure cultural appropriateness.

In previous studies, we created HPV vaccine reminder messages targeting parents to be sent via text message and postcards from their clinic [33,34,35,36]. Reminder educational messages followed the President’s Cancer Panel recommendations as well as parents’ preferences for motivational messages about HPV vaccination, which included a focus on the vaccine’s safety, ability to prevent six types of cancer, and importance of receipt at ages 9–12 [37,38,39,40,41]. For the current study, we adapted these messages using cultural targeting strategies to promote cultural appropriateness when targeting North Central rural-residing parents of unvaccinated 9- to 12-year olds (e.g., peripheral, constituent-involving, linguistic, and socio-cultural strategies) [42]. Using the same approach, we also developed new materials including a phone script for appointment reminders as well as promotional materials (text and flyer) informing families of the opportunity to vaccinate their adolescent at a mobile clinic in their community.

We aimed to further ensure these two evidence-based strategies (message reminders and mobile clinic implementation) appropriately targeted rural-residing parents in North Central Florida by soliciting parents’ perceptions on the mode as well as the design, content, and presentation of the materials (integrating their perspectives in our peripheral, linguistic, constituent-involving, and sociocultural strategies). We sought to identify: (1) message features that promote North Central Florida rural-residing parents’ receptivity to HPV vaccination; (2) parents’ acceptability of three reminder message modalities (text, postcard, and phone call); and (3) implementation factors that promote parents’ acceptability of using a mobile clinic for their child’s HPV vaccination. 

## 2. Methods

To explore parents’ preferred message features and identify factors critical for implementation, we used an interpretive qualitative design with interview methodology (individual and focus group) to ensure parents’ voices were at the forefront of the findings. This exploratory approach ensures the intervention can be targeted based on the population’s preferences.

### 2.1. Recruitment

Upon receiving IRB approval, we recruited parents for a focus group or individual interview from June 2023 to August 2023. The inclusion criteria for study eligibility were (1) being a parent of a 9- to 12-year-old whose child had not received the HPV vaccine, (2) residing in a rural North Central Florida county with a rural–urban continuum code ≥ 4 (i.e., Bradford, Columbia, Dixie, Hamilton, Lafayette, Levy, Madison, Putnam, Suwannee, Taylor, and Union), (3) being willing to receive study materials (e.g., the text messages, postcard, flyer, etc.) via their cell phone during the interview to provide feedback in real time, and (4) being able to speak and read English. 

The study recruitment material was disseminated using a multi-channel approach that included online ads via social media and a university study advertisement page for researchers. We also partnered with community partners including community health educators from the Institute of Food and Agricultural Sciences Extension to recruit parents in person at relevant local events (e.g., a public library, summer camps, 4H networks, a newspaper press release, and back-to-school events) and community pediatric clinics. Community partnerships in recruitment are critical in cancer prevention and control studies, particularly in reaching marginalized populations like rural-residing families [43]. Promotion materials included a link to a REDCap screening survey parents could complete to confirm eligibility or request follow-up from the study coordinator for more information. Eligible parents were asked to choose or provide available focus group interview times. At in-person recruiting events, parents were also able to sign up to be contacted about participating. The study coordinator contacted all interested parents to confirm eligibility and schedule participation. Eligible participants were offered a choice of in-person or online groups. Online focus groups allowed parents living far from the chosen locations to participate more easily. In-person focus groups accommodated parents who may not have access to the internet at home—among adults living rural areas of the United States, 87% have a smartphone and 76% have broadband [44].

Once parents chose an available time, they were directed to the consent form in REDCap. The consent information included that, if agreeing, they would take part in a group discussion with three to eight other parents of 9- to 12-year-olds. The discussion would include their opinions on a variety of proposed messages that their child’s clinic could send them about the HPV vaccine. Additionally, they would be asked to complete a brief background survey with questions about their child’s vaccine history. Following their consent, the parents were directed to complete the background survey. After completing the survey, the parents were sent instructions for Zoom. 

### 2.2. Procedures

A qualitative health behavior scientist [C.F.] led the development of a semi-structured guide to capture the parents’ perspectives of the two evidence-based strategies that can promote HPV vaccination. To ensure methodological congruence and cultural appropriateness, the interview guide was co-developed and reviewed multiple times by members of the multi-disciplinary team [C.F., C.B., A.F., M.H., S.S., M.V.], who have community-based, public health, and health communication expertise in developing study materials specifically within rural North Central Florida as well as for marginalized, diverse populations. The focus group interviews were moderated by a qualitative methods investigator with expertise in interviewing culturally diverse populations [A.F.]. Two parents who could not attend one of the scheduled focus group interviews were offered individual interviews.

Interviews and focus groups were conducted over a secure Zoom platform. While originally planned to hold in-person and Zoom-based focus groups, we only offered online focus groups because 100% of eligible parents chose online options of available dates and times. The study coordinator [M.H.] assisted the moderator by managing the audio-recording and sharing materials that included (1) a text message reminder of the child’s appointment to be sent two weeks before their birthday, (2) a mailed postcard reminder of the child’s appointment, (3) a phone call script of a clinician calling the parent, (4) a text about the mobile clinic, and (5) a flyer about the mobile clinic (See Supplementary Materials). For visual consistency with the postcard, which will include English and Spanish text, the postcard shared in the focus groups had both languages. The materials were shared with the participants via a text message and presented on the Zoom screen. When the phone script was shared, in addition to the above, we played an audio recording of actors playing the parts of the clinic staff and parent. The order of the materials changed each interview to reduce priming effects, though messages about reminders were presented together (birthday text, postcard, phone call script). The parents were asked to give feedback on each message, including their acceptability and preferences related to design and content. For the reminder (birthday) text message, they were also asked to rate (on their phones) how likely (on a five-point scale) they would be to respond “Yes” to then facilitate discussion about message features that promoted their receptivity about HPV vaccination. The moderator summarized the ratings and then asked the parents to explain their rating. The same approach was used for the phone call reminder script (i.e., asked to rate how likely they would be to schedule an appointment). The parents were also asked to rate which reminder message approach (text, postcard, phone call) they most preferred. They were also asked for feedback on mobile clinic promotional messages (a text and flyer) and their willingness (or lack thereof) to use a mobile clinic for vaccination. Upon completion, the parents were given a USD 60 e-gift code via email to compensate them for their time. 

### 2.3. Data Analysis

The transcripts were thematically analyzed using a constant comparative method (CCM) approach [45]. The analysis was led by a thematic analysis expert [D.M.], overseen by the qualitative methodology expert [C.F.], and validated by a separate coder trained by the lead methodologist to increase rigor [46]. The analysis involved three systematic steps conducted to identify typologies: (1) assigning codes (i.e., labels) to identify concepts in the text, (2) collapsing codes into categories to identify themes and the extent of saturation, and (3) conducting axial coding (i.e., finding patterns identified within the data specific to each theme) to characterize (i.e., define) each theme [45]. Repetition (repeated similar words to describe the same phenomenon), recurrence (using different terms to describe the same phenomenon), and forcefulness (emphasis) were the criteria used to identify the extent of thematic saturation [47]. Best practices in focus group methodology were also utilized by ensuring saturation was obtained across groups (i.e., interviews) and participants [48,49]. Saturation in findings was evident after four focus group interviews and confirmed in the fifth, which is in line with the best practices of sample sizes in implementation science focus group studies [50]. Both themes and the properties of each theme emerged in at least two focus groups and both interviews, with most found in three–five focus groups. To maintain confidentiality and include additional context, data exemplars are identified by individual interview or focus group numbers (INT1 or FG1), participant numbers in focus groups (P1), and if the parent’s 9- to 12-year-old child was a son or daughter.

## 3. Results

A total of 28 parents of 9- to 12-year-olds participated. Of these, 26 parents participated in one of five focus group interviews (3–8 parents in each group), and two parents participated in in-depth, individual interviews. The parents resided in five different rural counties in Florida. More than half (54%) had a two-year college degree or more. The parents identified as White (46%), Black (36%), or Hispanic (18%) (see Table 1). This was a more diverse representation relative to the ethnic and racial composition of these counties in North Central Florida, which is 8% Hispanic, 71% White, and 16% Black [5]. The parents had differed in the perception of the likelihood of their youngest 9- to 12-year-old child receiving the HPV vaccine in the next 12 months (47% likely, 39% unlikely, and 14% unsure).

The focus group interviews lasted, on average, 69 min (range = 57–77). The two interviews were, on average, 67 minutes (64–70 range). Audio recordings were professionally transcribed, resulting in 150 single-spaced pages of data.

### 3.1. Message Features That Promote Parents’ Receptivity to HPV Vaccination

The parents identified three message features (*source credibility*, *specific information coverage,* and *personalization with name and birthday*) that enhanced their receptivity to HPV vaccination messages regardless of how the message was disseminated (text, phone, postcard, or flyer). Thematic properties (in italics below) further define each message feature by capturing why it promoted the parents’ receptivity to the HPV vaccination message as well as parents’ recommendations for addressing the feature in message design.

#### 3.1.1. Feature 1: Source Credibility

The parents stressed the importance of knowing the message was from a credible source and described two ways to promote message credibility. They preferred messages *from a reputable, trustworthy source*. The parents specifically described trusting their child’s pediatrician or health clinic, reasoning that the reputation or credibility of the source recommending vaccination was influential: “I feel like people are really not rushing to get vaccines unless it came from your doctor. So, I think that [source] should be magnified” (Parent of daughter, FG2P2). Parents also recommended the *credible source be clearly visible to grab parents’ attention*. They recommended increasing the size of logos or names to grab their attention and promote their familiarity to enhance the likelihood that they would read the message:

[On the postcard] the [clinic] logo, maybe either bigger or in the front, because … things go straight in the trash. But sometimes it’s just like a normal reaction of the brain when you see something on there that’s familiar. Then you’re like, “Oh! What is this?”

(Parent of daughter, FG4P1)

#### 3.1.2. Feature 2: Specific Information Coverage

The parents described the importance of detailed information that informed their decision making and identified three specific areas of content to include on HPV vaccination messages.

They stressed the importance of *logistical vaccination information to promote feasibility.* This content included where, when, and how (e.g., scheduling options) to receive the vaccine, as well as the cost (i.e., that it was free), as that promoted parents’ awareness that it was “affordable and it’s available” (Parent of son, FG1P2). The parents also wanted *clear vaccine dosage information*. They acknowledged a lack of understanding or clarity about the number of vaccinations and timing:

It doesn’t [say] anywhere on the text or pamphlets about this being something that required more than one dose, or if it did, maybe I missed that part, where it’s like, “It could be two doses, or if you wait, there’s three doses involved.”

(Parent of son, INT2)

Finally, the parents wanted *additional, easily accessible, vaccine-related information*, including about side effects or HPV. They wanted the messages to include hyperlinks, QR codes, or phone numbers so that the parents could easily attain additional credible information about HPV vaccination they needed to answer their decision-making questions. They also linked having access to this information to promoting feasibility:

[During the phone call] they should say, “We have a whole web site where you can go to learn more about this. Would you like me to text that link to you?” Just make it easy. I mean, we’re already super busy and overloaded as parents go.

(Parent of son, INT1)

#### 3.1.3. Feature 3: Personalized Features

The parents also liked personalized features in the messages and provided two explanations about why this feature was important and could be promoted in the design. They shared that a *personalized message was attention-getting.* Using their child’s name or clinic on the text message or postcard, for instance, made the message feel personalized and grabbed their attention:

The personal touch helps [on the postcard]. A lot of times you get flyers and kind of that junk mail stuff in there, so, having something where it’s coming from our pediatrician’s office or has my kid’s name on it or something like that, then it might make me pause and be like, “Oh, wait a minute! This is actually specifically meant for me.” It’s not just a random flyer.

(Parent of son INT2)

The parents also explained how *certain message channels/modalities feel more personal,* specifically highlighting the text message and phone call received from their clinic. A parent shared, “When you get a call from your doctor, it’s like, ‘Oh, yeah. Okay. You told me to, I’ll do it.’ You trust them. When they call and it’s that personal phone call, it’s different” (Parent of son, FG2P3).

### 3.2. Parents’ Messaging Modality Preferences

While parents expressed being receptive to all three reminder message channels (text, postcard, phone call from pediatrician’ office), they preferred the text approach the most. The following parent juxtaposed how their receptivity to the messages was different based on the message modality used:

The phone call feels like you need to take action immediately and the postcard is too easy to not take any action because it would be thrown away or you set it down on the counter and then you don’t see it again. You don’t think about it. Whereas the text message, when you open your phone to go send a text, you’re still going to see. Oh, hey, there was that text message.

(Parent of son, FG4P4)

The parents further explained what they liked and did not like related to each message modality, which further reflected the message features they perceived as promoting receptivity.

#### 3.2.1. Modality 1: Text Message

The parents liked that the text was personalized with their child’s name and included birthday wishes. They also liked that the text included the specific information coverage they needed to make a decision (i.e., the option to click a link for further vaccination information) as well as what to do next (i.e., the option to receive a call to schedule an appointment). They also discussed how the text was easily accessible because it was on their phone, and they would likely see it again. They described this as helpful given their busy lifestyle as parents. 

#### 3.2.2. Modality 2: Postcard

The parents described the mailed postcard as acceptable but easily forgotten or ignored, which they tied to the norms of receiving a lot of junk mail. To promote receptivity, they suggested enhancing the visibility of the credible source on the postcard (i.e., their clinic) to draw their attention and increase the likeliness that they would read it from the mail. 

#### 3.2.3. Modality 3: Phone Call

The parents were the least receptive to the phone call script. While they described it as a more personalized approach (which promoted trustworthiness of the recommendation), they also characterized the script as “pushy.” They wanted more time to make a decision, with an option to call back and schedule an appointment, as opposed to making an appointment at that point in time. The parents also wanted the clinician to provide parents with a way to obtain further information (e.g., telling them about a web site to use, sending a text with a link before or during the call).

### 3.3. Factors That Promote Parents’ Willingness to Have Their Child Receive HPV Vaccination at a Mobile Clinic

While the parents were receptive to HPV vaccination at a mobile clinic, they also voiced concerns related to the mobile clinic being unfamiliar as well as potentially unnecessary if their child had a pediatrician. They described three factors to emphasize in the implementation and promotion of the mobile clinic to encourage parents’ willingness to use the mobile clinic: (1) *prioritize convenience and feasibility*, (2) *promote trustworthiness*, and (3) *provide detailed information*. 

#### 3.3.1. Factor 1: Prioritize Convenience and Feasibility in Implementation

The parents acknowledged that obtaining a vaccination at a mobile clinic could be convenient, which would enhance their willingness to use it. They suggested three factors to prioritize when implementing the mobile clinic. The parents wanted the clinic at a *convenient location*. It was important that it be geographically close (which may also mean at a community event they planned to attend). Some parents noted how the convenience of the mobile clinic could reduce barriers to HPV vaccination:

I think if I were in a situation where it was difficult to get to my child’s health care provider, whether transportation wise or distance from them, that would probably be the number one reason for me to do a mobile clinic.

(Parent of daughter, FG1P1)

The parents stressed that the mobile clinic be *offered during flexible times*. Specifically, the parents wanted it available during evening hours and weekends—times when their pediatrician’s office was not open. They noted the importance of “weekend hours for working parents” “because you don’t want [your child] to be missing school” or parents having to “tak[e] off work.” Finally, the parents thought *scheduling options* promoted convenience and wanted to be able to schedule an appointment in advance:

There should be appointments where you don’t have to be sitting outside just waiting or anything like that because that could discourage the kids [who] might not want to hang out and things like that. Have appointments and where you can get people in and out with no problems.

(Parent of daughter, FG5P3)

#### 3.3.2. Factor 2: Promote Trustworthiness in Implementation

The parents admitted being unfamiliar with mobile clinics, which inhibited their receptivity, but emphasized three implementation factors critical to enhancing their trust. The parents noted the importance that the mobile clinic be *staffed with credible/known clinicians*. This included clinical staff they were familiar with or staff from their local or well-known health institutions, particularly their child’s clinical provider, to promote parents’ trust and sense of familiarity:

I don’t mean to be complicated on the mobiles. … It’s just my lack of experience. They always feel a little too non-personal because it’s just business, you know what I mean? So, it feels a little less personal. … I guess it would be less personal just because it wouldn’t be people that we know or a place we’ve ever been. So, there’s just not that familiarity where it feels safe and comfortable in that sense because it does feel more kind of like a weird transaction.

(Parent of son, INT1)

The parents also shared that they would trust the mobile clinic if their *pediatrician recommended or endorsed it*:

That would be the biggest thing for me would be if my healthcare provider said, “This is coming to your area. I know these people. I trust them. I recommend that you get your vaccine there.” That would probably be, I would say, the first and biggest reason that I would do something like that.

(Parent of daughter, FG1P1)

The parents also shared that they would find the mobile clinic more trustworthy if it was *at a trusted community location*. They specifically mentioned having a mobile clinic at their local church or school, which enhanced credibility and familiarity: “I know there’s a lot of mobile clinics that do the blood banks at the churches, and they do get a lot of people that’ll come to them because it’s at the church” (Parent of daughter, FG2P3).

#### 3.3.3. Factor 3: Provide Detailed Information in Promotion

The parents explained how having specific information covered in the promotional materials promoted their receptivity to doing their child’s vaccination at a mobile clinic. This included addressing two issues in the promotion of the mobile clinic. The parents wanted *the how-to’s* of mobile clinic vaccination. They wanted to know “why” they should use it, “how” to use it (e.g., vaccine scheduling and dosing information), and “where/when” they could use the mobile clinic (e.g., time/location, contact information). When viewing the mobile clinic text message, a parent said:

I like the message because they have the date that we’re supposed to do the vaccination. And they got the time, they got the address, phone number, they got most of it, the whole information that we need to make an appointment.

(Parent of son, FG1P3)

The parents also wanted access to *more information about the HPV vaccine*, including side effects and effectiveness, via a link or QR code. One parent explained further research would help in their decision making: “I’m the type that before I allow my child to get a vaccine, I’m going to research it. I want to know the pros, the cons, everything” (Parent of son FG3P1).

## 4. Discussion

An ethnically diverse group of parents of 9- to 12-year-olds not currently vaccinated against HPV living in rural, North Central Florida provided feedback to ensure two evidenced-based strategies (reminder messages to inform/prompt vaccination appointments and mobile clinics to reduce transportation barriers) were culturally targeted to increase the participation of their children in HPV vaccination. The parents’ feedback supported the notion that vaccination reminder messages and mobile clinics are acceptable evidence-based strategies for use among rural-residing parents, as they enhance their ability to follow through with vaccination by promoting parents’ awareness of the importance of HPV vaccination for their children (especially through text message reminders) and by increasing vaccination access (e.g., through flexible appointment times and mobile clinics). At the same time, the parents provided keen insights on how to enhance these evidence-based strategies prior to implementation (focusing specifically on trust and feasibility) to maximize rural-residing parents’ HPV vaccination uptake and follow-through.

Institutional and interpersonal trust are known factors associated with vaccine hesitancy [36,51,52]. Similar to a study regarding COVID-19 vaccination in rural regions of the United States [53], the parents described that to be effective in increasing vaccination, materials must generate trust, and they already trust their local communities and children’s doctors. Focusing vaccine interventions on components that promote parents’ trust is critical to influencing behavior and can include community partnerships, autonomy-enhancing approaches, and targeted risk information [54]. The parents in our study provided specific suggestions about how to promote parents’ trust of the two evidence-based strategies that complemented the literature, which may enhance parental interest and involvement in HPV vaccination programs. For instance, they described being receptive to HPV messaging regardless of modality (via phone, text, postcard) when they trusted the source of the message (which was their child’s healthcare clinic or pediatrician). The parent acceptance of all modes of vaccination delivery suggests that these modes may have similar effectiveness at increasing vaccination, as evidence suggests preference may be more important than mode; however, their feedback related to phone call hesitancy (not feeling pressured/pushy) and postcards (not appearing as general bulk mail) should be accounted for in the design of the materials [55]. The parents also described being receptive to HPV vaccination offered by a mobile clinic if it was staffed by people they trust (e.g., clinicians from their local healthcare system or clinic), located in places they trust (e.g., their child’s school or local churches), and referred by people they trust (e.g., their child’s pediatrician).

The parents’ acceptability of the content of vaccine messages can be enhanced when providing information that makes the messages seem personal. These findings are consistent with the broader focus on promoting healthy behaviors with personalization, including identifying information to make the message unique or tailoring the message content [56,57,58]. Our study adds to prior research indicating that parents find vaccine messages more acceptable when the child’s name is included by expanding this finding to rural areas and timing the message around the child’s birthday rather than providing the child’s date of birth, which is sensitive information [36,59].

Vaccine-hesitant rural community members have described barriers to accessing information about vaccination as a critical issue in their hesitancy to follow through with vaccination [53]. The parents in our study expressed that they wanted access to vaccination information (via a QR code or link) via all message modalities—when reminders were sent via text, phone, or postcard—to inform their decision making. This finding of parents’ desire for more information is supported by the literature indicating that vaccine hesitancy is more likely among those who feel frustrated in their efforts to find vaccine information and that messages providing information on vaccine side effects and efficacy can increase trustworthiness and intentions to vaccinate [60,61,62]. Moreover, vaccine reminders that include educational information or an opportunity to link to educational information are more effective than reminders without this information [63]. Enhancing access to HPV vaccination information is especially critical given that rural-residing parents, including those living in rural Florida, are reported to have less knowledge about HPV vaccination [64,65].

Mobile clinics, when implemented in a manner that promotes parents’ trust and vaccination feasibility, may be an ideal bridge for rural-residing parents and promote their follow-through with HPV vaccination. It may also serve as a vehicle for HPV education dissemination, thereby ensuring parents have access to the vaccination and the information needed to inform their decision making. While not widely studied, mobile clinics have been advocated for recently in the wake of the COVID-19 pandemic but also for sustaining this practice to reach underrepresented groups and traditionally hard-to-reach marginalized populations like migrant and seasonal farm worker families in rural regions [66,67,68,69]. The parents in our study endorsed the importance of mobile clinics, and in line with other related studies, noted that this approach can decrease vaccination access barriers if offered during weekends and evening hours [70]. However, the parents also acknowledged hesitancy tied to a lack of familiarity with mobile clinics or a sense that it was unnecessary if they had a pediatrician. Their trust in the mobile clinic and resultant vaccine uptake may be facilitated by promotional materials from community sources they trust that also highlight the feasibility of vaccinating at a mobile clinic to increase parents’ follow-through.

There are several limitations to this study. While our unit of analysis (number of interviews) was strong for focus group design in implementation science [49,50], our overall sample was limited. Our findings may be transferable to similar populations living in rural, southern regions of the country where vaccination is low and parents’ trust is a factor in HPV vaccination for their children. Our participants identified mostly as White non-Hispanic. While only 18% identified as Hispanic and 36% identified as Black non-Hispanic, this is more diverse than the composition of the North Central Florida counties, which are 8% Hispanic, 71% White, and 16% Black [5]. The entire state of Florida’s population is 53% Hispanic [71]. Thus, while Hispanic populations are less prevalent in North Central Florida [71], the present sample could limit the transferability of our findings to more racially and ethnically diverse rural-residing parents in other regions of the state. In addition, our sample represented English-speaking parents, which may not transfer to non-English-speaking parents in the same region. Future studies should also explore additional cultural factors related to race and ethnicity to further culturally target message features when addressing population subgroups [9,42].

Our study design could be replicated in other studies aimed at culturally targeting evidence-based strategies for intervention implementation with more diverse populations. Technology is also important to consider in the study design to capture parents’ perceptions. In the current study, the parents had to be willing to receive text messages (of the reminder messages and to rate them) during the interview (which was advertised in the recruitment materials). Although our participants did not encounter technical challenges or express concerns/frustrations during the interviews, parents unwilling to receive text messages via their phones may not respond to initial recruitment requests.

## 5. Conclusions

This study demonstrates the importance of sharing intervention materials and approaches with the targeted community to promote cultural appropriateness in the intervention design to enhance parents’ receptivity to evidence-informed strategies (reminder messages and mobile clinics) focused on increasing HPV vaccination among 9- to 12-year-olds. Our findings suggest that during the post COVID-19 vaccine era, parents remain concerned with the trustworthiness of vaccine messages and vaccine providers. Both of the evaluated evidence-based strategies (reminder messages and mobile clinics) are acceptable to parents from diverse racial/ethnic backgrounds living in rural areas of Florida. 

## Figures and Tables

**Table 1:** Participant Characteristics.

Relationship to Child	
Biological/Adoptive parent	25 (89.3%)
Aunt or Uncle	3 (10.7%)
Marital Status	
Never Married	7 (25%)
Married	15 (53.6%)
Divorced/Separated/Widowed	6 (21.4%)
Gender	
Female	26 (92.8%)
Male	1 (3.6%)
Unknown	1 (3.6%)
Race/Ethnicity	
Non-Hispanic White	13 (46.4%)
Non-Hispanic Black	8 (28.6%)
Hispanic or Latino Origin	5 (17.8%)
Black or African American w/Unspecified Hispanic or Latino Origin	2 (7.2%)
Level of Education	
Graduate degrees (PhD, EdD, MD, DDS, DVM, JD, MBA, MA)	5 (17.8%)
Bachelor’s Degree	6 (21.4%)
Associate’s degree	4 (14.3%)
Some college credit or trade school	4 (14.3%)
High school graduate/GED	8 (28.6%)
9–12th grade, no diploma	1 (3.6%)
Number of 9- to 17-year-olds	
1 Child	14 (50%)
2 Children	6 (21.4%)
3 Children	5 (17.9%)
5 Children	3 (10.7%)
Age of Youngest 9- to 12-year-old	
9-year-old	10 (35.8%)
10-year-old	7 (25%)
11-year-old	5 (17.8%)
12-year-old	6 (21.4%)
Gender of Youngest 9- to 12-year-old	
Female	15 (53.6%)
Male	13 (46.4%)
Number of Well Visits in Past 12 Months	
0 visits	4 (14.3%)
1 visit	15 (53.6%)
2 or more visits	9 (32.1%)
Delayed Vaccine of Youngest 9- to 12-year-old Child Due to Reasons Other than Illness or Allergy	
Yes	8 (28.6%)
No	19 (67.8%)
Did not answer	1 (3.6%)
Decided Not to Have Youngest 9- to 12-year-old Child Get a Vaccine for Reasons Other Than Illness or Allergy	
Yes	7 (25%)
No	20 (71.4%)
Don’t know	1 (3.6%)
Likelihood of Youngest 9- to 12-year-old Child Receiving the HPV Shots in the Next 12 Months	
Very likely	3 (10.7%)
Somewhat likely	10 (35.7%)
Not too likely	3 (10.7%)
Not likely at all	8 (28.6%)
Not sure/Don’t know	4 (14.3%)
Youngest 9- to 12-year-old Child Received the Following Vaccines	
MenACWY and Tdap	6 (21.4%)
Tdap only	11 (39.3%)
I don’t know	10 (35.7%)
Did not answer	1 (3.6%)

## Data Availability

Data presented in the study were not approved by IRB for open access. Requests can be made to the corresponding author to seek approval for sharing data.

## Supplementary Materials

The following supporting information can be downloaded at: https://www.mdpi.com/article/10.3390/vaccines12070712/s1.
