# Supplementary material for: Promoting Rural-Residing Parents’ Receptivity to HPV Vaccination: Targeting Messages and Mobile Clinic Implementation
Source: Vaccines (Basel). 2024 Jun 26;12(7):712. doi: 10.3390/vaccines12070712 (PMC11281438; doi:10.3390/vaccines12070712)

Text reminder

Kids Clinic: Happy birthday to Marta! We recommend that all children Marta's age receive the HPV vaccine. They can get the vaccine at any visit including their regular check-up. It is safe, free and can protect them from 6 types of cancers. To learn more, go to https://ufhealth.org/hpv Reply YES to have Marta's office call you to schedule an appointment. Reply STOP to opt-out. Msg&data rates may apply.

Postcard

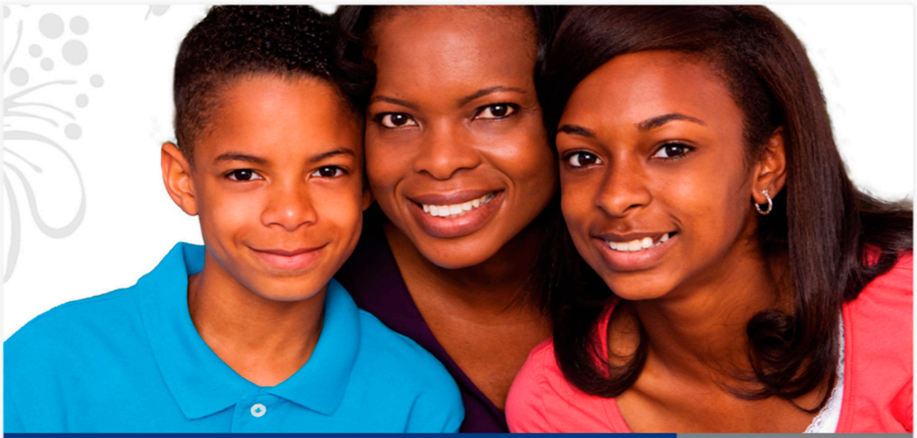

PROTECT YOUR CHILD FROM 6 TYPES OF CANCER

GET THE FACTS

PROTEJA A SU HIJO CONTRA 6 TIPOS DE CÁNCER

¡INFÓRMESE!

FACT: A virus called the Human Papillomavirus (HPV) causes 6 types of cancer.

FACT: We recommend the vaccine for ages 9 and up that protects against HPV and prevents 70% of these cancers.

FACT: The HPV vaccine is safe.
Side effects are similar to other vaccines and may include: headache, fever, and pain or swelling at the injection site.

FACT: The vaccine works best when received at ages 9 to 12 years.

[Name] can get the HPV vaccine for FREE.
[Nombre] puede obtener la vacuna contra el HPV de manera GRATUITA.

DATO: Un virus llamado "el virus del papiloma humano" (HPV, por sus siglas en inglés) causa 6 tipos de cáncer.

DATO: Recomendamos la vacuna a partir de los 9 años de edad que protege contra el HPV y previene 70% de estos cánceres.

DATO: La vacuna contra el HPV es segura.
Los efectos adversos son similares a los de otras vacunas, como dolores de cabeza, fiebre, dolor e hinchazón en el sitio de aplicación de la vacuna, entre otros.

DATO: La vacuna funciona mejor cuando es administrada entre las edades de 9 a 12 años.

UF UNIVERSITY of FLORIDA

ufhealth.org/hpv
555-555-5555

Kids Clinic

You have received this postcard because the University of Florida is working with Kids Clinic to improve adolescent vaccination rates.
Usted ha recibido esta postal porque la Universidad de la Florida está trabajando con Kids Clinic para mejorar los índices de vacunación entre los adolescentes..

Phone call script

Phone call script:
Clinic staff: Hello, Ms. Parker. This is Sara from Kids Clinic. We are calling to remind parents about needed vaccines. Before we get started, how is Blake doing?
Parent: Blake is doing fine, and just started back in school after the holidays. He can't wait for soccer to start.
Clinic staff: That sounds great! I am glad Blake continues to get such great exercise from soccer. The reason I am calling is that I want to tell you that we have a vaccine for kids Blake's age that prevents against six types of cancer called the HPV vaccine. We are calling all parents of 9-year - olds to recommend that their child get this safe vaccine today. Once they get the first dose, they will come back in six months to get the second dose. Can we set up an appointment for you to bring Blake in to get the vaccine?
Parent: Oh. Thanks for calling. I don't know if we really have time with soccer starting.
Clinic staff: I understand. It is so hard at that age to find time in their schedules. If Blake starts the HPV vaccine now, we will only need to do two doses instead of three. We can schedule the second dose with his annual exam. We really recommend Blake gets this vaccine to prevent cancer. What about Tuesday next week after school?
Parent: Ok. I guess we can do it next week since soccer does not start until the week after. Then we can do the second dose at annual visit and we don't have to make a special trip.

Mobile clinic text

Kids Clinic: Partnering with the University of Florida, we are happy to offer adolescents vaccines in our community. Marta can receive any needed vaccines free of charge at Community Fair on October 20, 2023 from 9 am to 5 pm. This is a great chance to get the second dose of the HPV vaccine so they can be protected from 6 types of cancers. To schedule an appointment, go to weblink, call 352-627-9075, or just drop by at 2300 N Temple Ave, Starke, FL 32091.
Reply STOP to opt-out. Msg&data rates may apply.

Mobile clinic flyer

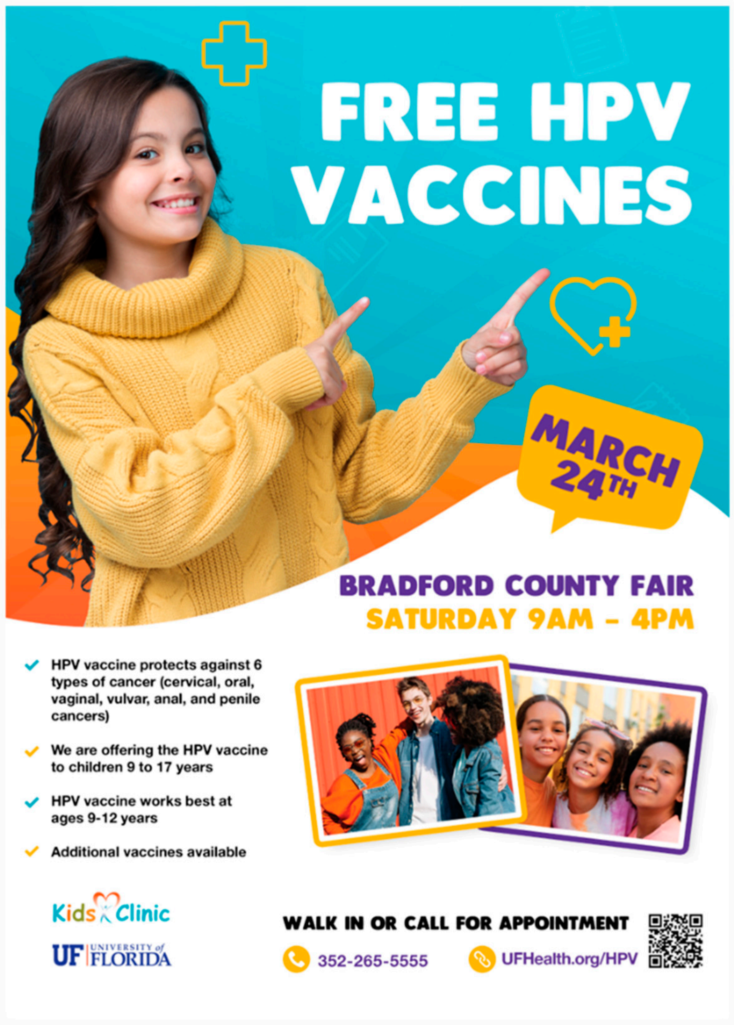

Supplement: Supplementary file 1 [file vaccines-12-00712-s001.zip › vaccines-3027830-supplementary.pdf]
